# G-Protein Coupled Receptor 18 Contributes to Establishment of the CD8 Effector T Cell Compartment

**DOI:** 10.3389/fimmu.2018.00660

**Published:** 2018-04-04

**Authors:** Hayakazu Sumida, Jason G. Cyster

**Affiliations:** Department of Microbiology and Immunology, Howard Hughes Medical Institute, University of California, San Francisco, San Francisco, CA, United States

**Keywords:** G-protein coupled receptor 18, CD8 T cells, effector-memory, KLRG1, granzyme B

## Abstract

The requirements for effector and memory CD8 T cell development are incompletely understood. Recent work has revealed a role for G-protein coupled receptor 18 (GPR18) in establishment of the intestinal CD8αα intraepithelial lymphocyte compartment. Here, we report that GPR18 is also functionally expressed in conventional CD8αβ T cells. When the receptor is lacking, mice develop fewer CD8^+^ KLRG1^+^ Granzyme B^+^ effector-memory cells. Bone marrow chimera studies show that the GPR18 requirement is CD8 T cell intrinsic. GPR18 is not required for T-bet expression in KLRG1^+^ CD8 T cells. Gene transduction experiments confirm the functional activity of GPR18 in CD8 T cells. In summary, we describe a novel GPCR requirement for establishment or maintenance of the CD8 KLRG1^+^ effector-memory T cell compartment. These findings have implications for methods to augment CD8 effector cell numbers.

## Introduction

CD8 T cells that have responded to antigenic stimuli have been classically divided into CD44^hi^ CD62L^lo^ effector memory (EM) and CD44^hi^ CD62L^hi^ central memory (CM) cells (1). Early studies on the CD8 T cell response following lymphocytic choriomeningitis virus (LCMV) and *Listeria* infection showed that CD8 T cells expand and differentiate through an early effector cell (EEC) stage into distinct effector populations, including short-lived effector cells (SLEC) and memory precursor effector cells (MPEC) (2, 3). SLECs are distinguished by high expression of KLRG1 and low expression of the IL7Rα chain (CD127), while MPEC have the reciprocal marker pattern (4, 5). Both types of cell express effector molecules such as Granzyme B and IFNγ, but only MPECs are efficient at giving rise to memory responses. Subsequent studies in a number of systems have shown a less clear correlation between expression of KLRG1 and a short-lived effector state. In some cases, the KLRG1^+^ cells persisted to the memory phase and provided effective control of the infection despite weak recall proliferative responses (6, 7). Other studies have noted that the amount of KLRG1 expressed by the effector-memory population may be determined by the amount of exposure to inflammatory signals during CD8 cell differentiation (8, 9). While all the factors responsible for determining the size of the KLRG1^+^ effector-memory population have not been defined, it has been established that the size of this compartment can be promoted by the pro-survival activity of IL-15 and restricted by the proapoptotic effect of TGFβ (4, 10). Several studies have shown a role for high expression of the transcription factor T-bet in establishing the KLRG1^+^ effector cell compartment (11–13).

The G-protein coupled receptor G-protein coupled receptor 18 (GPR18) is abundantly expressed in lymphocytes, with particularly high expression in CD8αα γδT intraepithelial lymphocytes (IELs) (14). Two recent studies using independently generated GPR18-deficient mouse lines found that this receptor plays a role in establishing an IEL compartment of normal size (14, 15). However, whether this receptor has functions in conventional T cells has been unknown.

In the course of our work to characterize how GPR18 contributes to IEL function, we noticed that GPR18-deficient mice had a lower frequency of CD44^hi^ CD62L^lo^ effector-memory type CD8 T cells. Here, we have characterized this deficiency and find that GPR18 knockout (KO) mice have lower numbers of spontaneously forming KLRG1^+^ CD8 effector-memory cells.

## Materials and Methods

### Mice, Reagents, and Infection

C57BL/6J (B6, CD45.2) and congenic B6 CD45.1^+^ mice were from the Jackson Laboratory, and these strains were intercrossed to generate B6 CD45.1/2 F1 mice. *Gpr18*^−/−^ mice were generated as described (14) To generate bone marrow (BM) chimeras, CD45.1^+^ B6 mice were irradiated by exposure to 1,100 rad of γ-irradiation in two doses 5 h apart and i.v. injected with at least 2 × 10^6^ total BM cells from each genotype of mice as indicated and analyzed after 2–3 months. All chimeras appeared healthy at the time of analysis. For LCMV infection, mice were infected with 2 × 10^5^ plaque-forming units of LCMV Armstrong administered i.v. Animals were housed in a specific pathogen-free environment in the Laboratory Animal Research Center at the UCSF, and all experiments conformed to ethical principles and guidelines approved by the UCSF Institutional Animal Care and Use Committee.

### Cell Preparations

For peripheral blood lymphocytes (PBL) preparation, blood was collected into EDTA-coated tubes and red cell lysis was performed. Splenocyte and mesenteric lymph node cell suspensions were prepared by mashing the organs through 70-µm cell strainers and then suspended with RPMI-1640 medium supplemented with 5% FCS.

### Antibodies and Flow Cytometry

Cells were stained using standard procedures for surface markers. The following monoclonal antibodies were used for flow cytome-try: TCRβ (H57; BioLegend), CD4 (GK1.5; BioLegend), CD8α (53.6.7; Tonbo Bio), CD8β (H35; eBioscience), CD45.1 (A20; BioLegend), CD45.2 (104; BioLegend), CD62L (MEL-14; BioLegend), CD44 (IM7; BD), KLRG1 (2F1/KLRG1; BioLegend), CD127 (A7R34; eBioscience), CXCR3 (CXCR3-173; BioLegend), IFNγ (XMG1.2; BD), Granzyme B (GB11; Invitrogen), Ki-67 (B56; BD), and T-bet (4B10; BioLegend). For cell sorting, dead cells were excluded using Fixable Viability Dye eFluor780 (eBioscience) and sorted splenocytes were more than 95% pure. For intracellular Granzyme B or T-bet staining, harvested cells were stained for surface markers then fixed and permeabilized with Cytofix/Cytoperm (BD) or Intracellular Fixation and Permeabilization Buffer Set (eBioscience), respectively. For intracellular IFN*-*γ staining, harvested splenocytes were stimulated with 100 ng/ml phorbol myristate acetate (PMA) and 1 µg/ml ionomycin for 4–5 h in the presence of GolgiPlug (Brefeldin A, BD). After culture, cells were stained for surface markers then fixed and permeabilized with Cytofix/Cytoperm (BD) and stained for intracellular cytokines. Flow cytometric analysis was performed using a BD LSRII. Sorting was with a BD Aria instrument. Flow cytometry data were processed using FlowJo version 10.2 software (Tree Star).

### Quantitative RT-PCR

Total RNA from sorted cells was extracted using an RNeasy kit (Qiagen) and reverse-transcribed. Quantitative PCR was performed as described (16). Data were analyzed using the comparative CT (2^−ΔΔCt^) method using *Hprt* as the reference. The primers were as follows:

*Hprt*: sense primer, 5′-AGGTTGCAAGCTTGCTGGT-3′ and antisense primer, 5′-TGAAGTACTCATTATAGTCAAGGGCA-3′.
*Gpr18*: sense primer, 5′-CTCTCTCTGGGACTGGGCAG-3′ and antisense primer, 5′-GGTGGCCATCTTACAGCAGG-3′.

### Retroviral BM Transduction

*Gpr18^−/−^* (CD45.2^+^CD45.1*^−^*) cells were retrovirally transduced with MSCV2.2 retroviral vectors containing GPR18 or empty vector and an IRES–GFP reporter as described previously (17). Virus was produced using PlatE cells grown in DMEM + 10% FCS + P/S + 10 mM HEPES + Q(Glu) (without P/S during transfection). BM cells were harvested 4 days after 5-flurouracil (Sigma) injection and cultured in the presence of recombinant IL-3, IL-6, and mouse stem cell factor (100 ng/ml, Peprotech). BM cells were spin-infected twice with a retroviral construct expressing GPR18 or empty vector and an IRES–GFP cassette as a reporter. One day after the last spin infection, cells were injected into lethally irradiated CD45.2*^−^*CD45.1^+^ B6 recipients.

### Intravascular Staining

A total of 3 µg anti-CD8α-PE (clone 53-6.7 from Biolegend) antibody was injected i.v. At 3 min after injection, the animals were sacrificed and analyzed as described (18).

### Statistical Analysis

Prism (GraphPad, ver. 5.0a) software was used for all statistical analyses. Two-tailed, unpaired Student’s *t*-tests were performed when comparing two groups. *p-*Values less than 0.05 were considered significant. In graphs, horizontal lines indicate means, and error bars indicate SEM.

## Results

**Figure 1:**
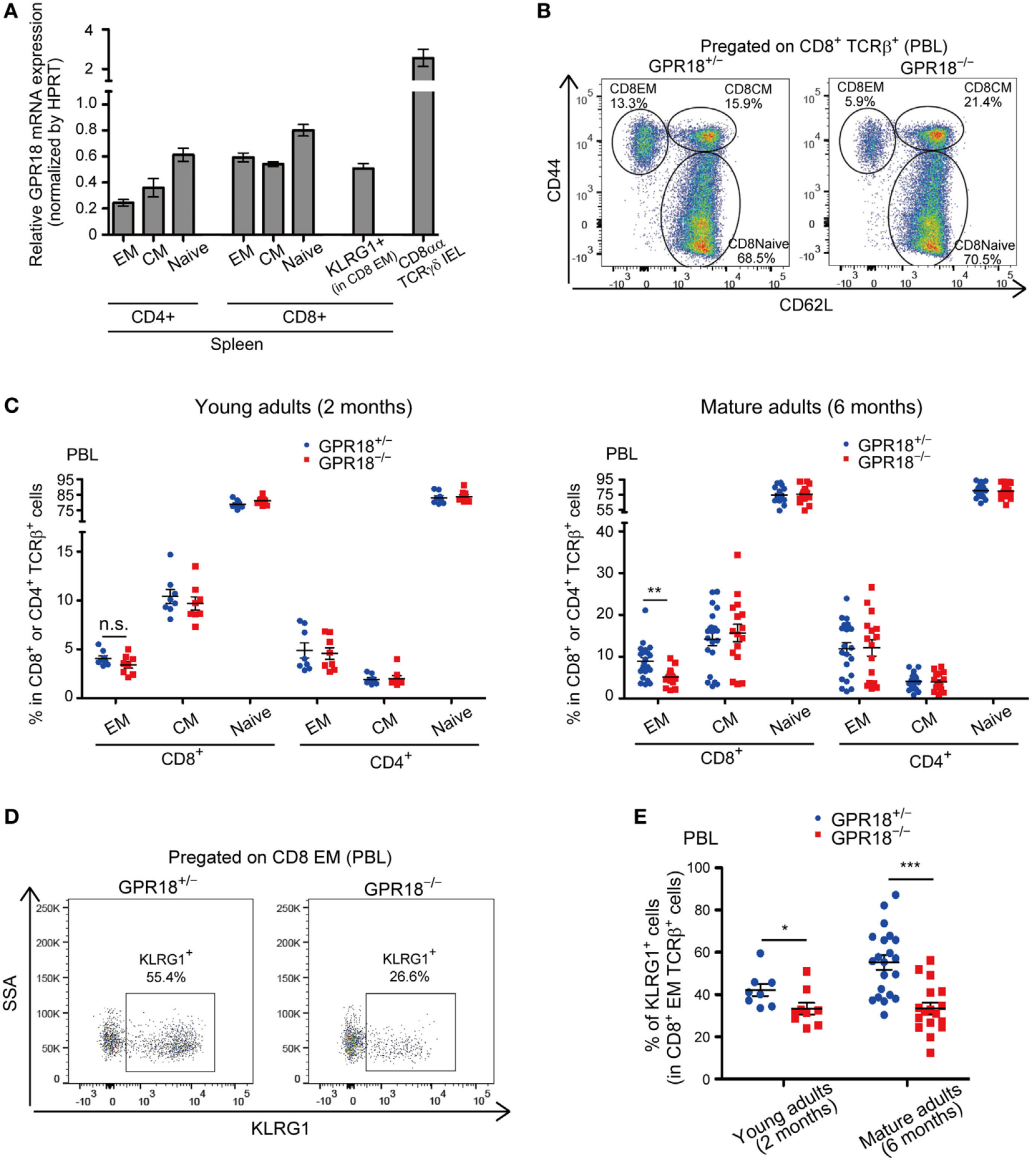
Reduction of KLRG1^+^ CD8 EM T cells in G-protein-coupled receptor 18 (GPR18)-deficient peripheral blood lymphocytes (PBL). **(A)** *Gpr18* transcript abundance in the indicated cell subsets relative to *Hprt*. Each point indicates cells sorted from an individual mouse and lines indicate mean ± SEM. *n* = 3 or 4 in each populations. EM, effector-memory; CM, central memory; IEL, intraepithelial lymphocytes. **(B)** Flow cytometric analysis of CD44 and CD62L expression in CD8^+^ TCRβ^+^ PBL from the indicated mature (6 months old) mice. Numbers show percentage of cells in the indicated gate. **(C)** Frequency of EM (CD44^hi^ CD62L^lo^), CM (CD44^hi^ CD62L^hi^), and naive (CD44^lo^ CD62L^hi^) CD8^+^ or CD4^+^ TCRβ^+^ cells in PBL from young (2 months old, left panel) or mature (6 months old, right panel) *Gpr18*^+/^*^−^*and *Gpr18^−/−^* mice. Left panel: *Gpr18*^+/^*^−^, n* = 8; *Gpr18^−/−^, n* = 8. Right panel: *Gpr18*^+/^*^−^, n* = 21; *Gpr18^−/−^, n* = 16. **(D)** Flow cytometric analysis of KLRG1 expression in CD8 EM PBL from the indicated mature (6 months old) mice. Numbers show percentage of cells in the indicated gate. **(E)** Frequency of KLRG1^+^ CD8 EM PBL in indicated young (2 months old) or mature (6 months old) mice. Young adults: *Gpr18*^+/^*^−^, n* = 8; *Gpr18^−/−^, n* = 8. Mature adults: *Gpr18*^+/^*^−^, n* = 21; *Gpr18^−/−^, n* = 16. Each point represents data from an individual mouse and lines represent means ± SEM **(C,E)**. ***p < 0.001, **p < 0.01, *p < 0.05, “n.s.” *p* > 0.05 by Student’s *t*-test **(C,E)**.

Analysis of GPR18 transcript expression in T cell subsets confirmed the high expression in CD8αα IELs and revealed considerable expression in CD8 T cells and slightly lower expression in CD4 T cells (Figure 1A). Expression was retained in CD44^hi^ CD62L^lo^ EM and CD44^hi^ CD62L^hi^ CM CD8 T cells. Flow cytometric analysis of CD44^hi^ CD62L^lo^ EM CD8 T cell frequencies in blood revealed a slight reduction in young (2-month-old mice) and a significant reduction in mature (6-month old) GPR18-deficient mice (Figures 1B,C). The frequencies of blood CD4 T cells and of naïve and CM CD8 T cells were unaltered in GPR18-deficient mice (Figure 1C; Figure S1A in Supplementary Material). Examination of marker expression within the CD8 EM compartment revealed a strong deficiency in KLRG1^+^ cells in the GPR18 KO mice (Figures 1D,E). Given that exposure to commensal and environmental antigens may differ between mouse cages, our studies were done with co-cocaged littermate mice generated in heterozygote by KO intercrosses. We do not exclude the possibility that GPR18 heterozygosity causes a partial effect on the CD8 compartment that may cause us to underestimate the magnitude of the KO phenotype.

**Figure 2:**
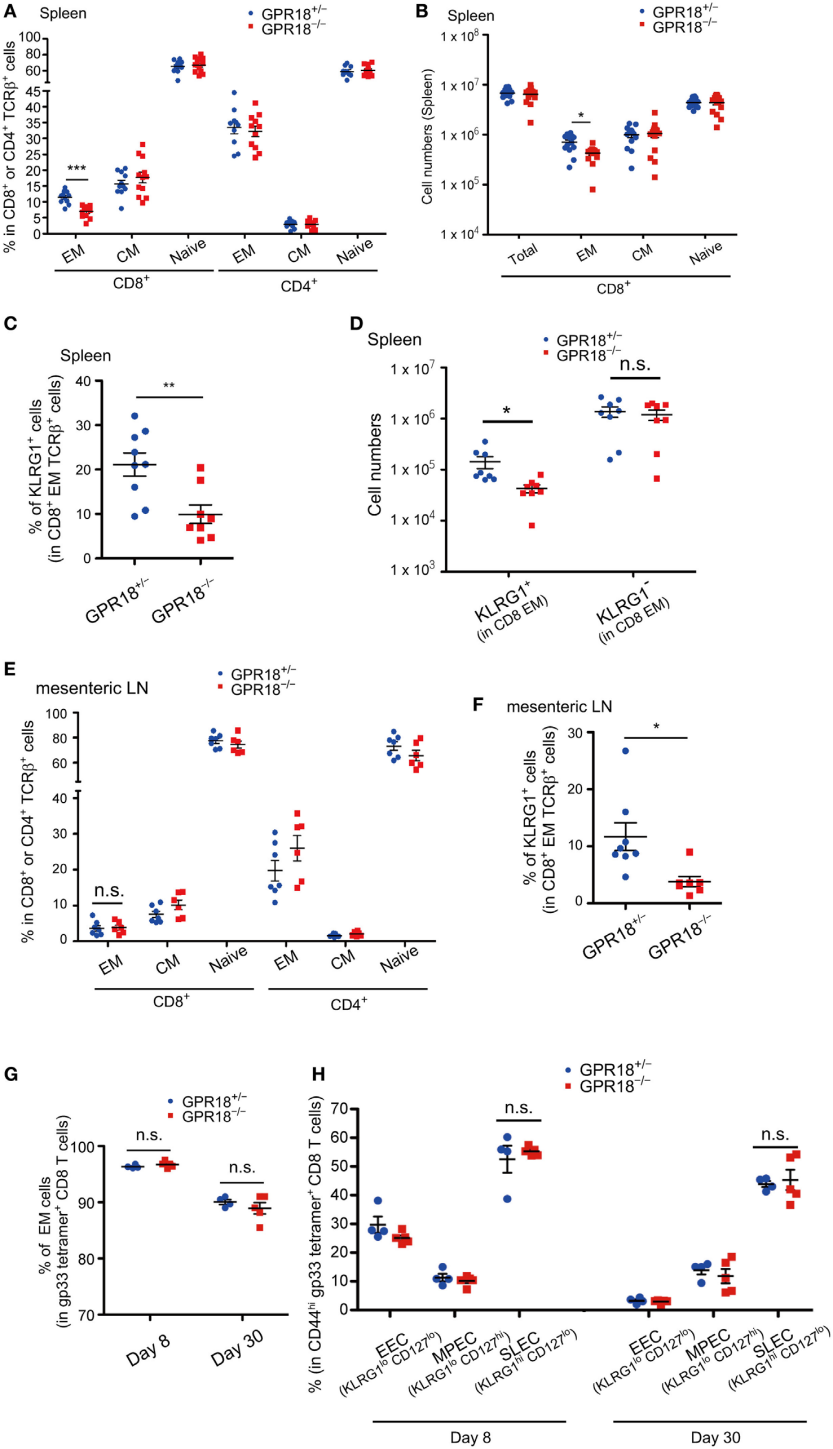
Reduction of KLRG1^+^ CD8 effector memory (EM) T cells in lymphoid tissues of G-protein coupled receptor 18 (GPR18)-deficient naïve mice **(A)** Frequency of EM (CD44^hi^ CD62L^lo^), central memory (CM) (CD44^hi^ CD62L^hi^), and naive (CD44^lo^ CD62L^hi^) populations in CD8^+^ or CD4^+^ TCRβ^+^ splenocytes in mature mice (6 months old) of the indicated type. *Gpr18*^+/^*^−^, n* = 11; *Gpr18^−/−^, n* = 12. **(B)** Number of CD8^+^ TCR β^+^ splenocytes in the indicated mature (6 months old) mice. Each population was pre-gated on CD45^+^TCRβ^+^ cells. *Gpr18*^+/^*^−^, n* = 13; *Gpr18^−/−^, n* = 14. **(C)** Frequency of KLRG1^+^ cells in CD8 EM splenocytes in indicated mature (6 months old) mice. *Gpr18*^+/^*^−^, n* = 9; *Gpr18^−/−^, n* = 8. **(D)** Numbers of KLRG1^+^ CD8 EM splenocytes in indicated mature (6 months old) mice. *Gpr18*^+/^*^−^, n* = 8; *Gpr18^−/−^, n* = 8. **(E)** Frequency of EM, CM, and naive populations in CD8^+^ or CD4^+^ TCRβ^+^ lymphocytes from mesenteric lymph nodes (mLN) in indicated mature (6 months old) mice. *Gpr18*^+/^*^−^, n* = 7; *Gpr18^−/−^, n* = 6. **(F)** Frequency of KLRG1^+^ populations in CD8 EM from mLN in indicated mature (6 months old) mice. *Gpr18*^+/^*^−^, n* = 8; *Gpr18^−/−^, n* = 7. **(G)** Percentages of *Gpr18^−/−^* or control *Gpr18^−/−^* EM cells in gp33 tetramer^+^ CD8 T cells in peripheral blood lymphocytes (PBL) at day 8 and day 30 after lymphocytic choriomeningitis virus (LCMV) Armstrong infection. **(H)** Percentages of early effector cell (EEC) (KLRG1^lo^ CD127^lo^), memory precursor effector cells (MPEC) (KLRG1^lo^ CD127^hi^), and short-lived effector cells (SLEC) (KLRG1^hi^ CD127^lo^) in gp33 tetramer^+^ CD44^hi^ CD8 PBL at day 8 and day 30 after LCMV Armstrong infection. **(G,H)** *Gpr18*^+/^*^−^, n* = 4; *Gpr18^−/−^, n* = 5. Each point represents data from an individual mouse and lines represent means ± SEM **(A–H)**. ***p < 0.001, **p < 0.01, *p < 0.05, “n.s.” *p* > 0.05 by Student’s *t*-test.

The reduction in CD8 EM cells, but not CD4 EM cells, was also observed in the spleen of mature-aged mice (Figure 2A; Figure S1B in Supplementary Material). Enumeration of total spleen cells established that there was an overall deficiency in EM CD8 T cells (Figure 2B). Staining for KLRG1 confirmed the selective deficiency of KLRG1^+^ cells amongst CD8 EM cells (Figures 2C,D). A deficiency in KLRG1^+^ CD8 EM cells was also observable in mesenteric LNs (Figures 2E,F). Regarding GPR18 expression, KLRG1^+^ and KLRG1*^−^* CD8 EM cells showed comparable mRNA levels (Figure 1A).

The KLRG1^+^ CD8 EM cells studied above were those arising endogenously in mice housed in our specific pathogen-free mouse facility. To test whether GPR18 was involved in the generation of KLRG1-expressing cells that appear rapidly following viral infection, GPR18 KO and control mice were infected with LCMV Armstrong and examined after 8 and 30 days. Gating on LCMV-specific gp33-tetramer^+^ CD8 T cells revealed an equivalent percentage of EM cells in control and GPR18 KO mice (Figure 2G; Figure S2 in Supplementary Material). Gating on total CD44^hi^ gp33-tetramer^+^ cells showed an equivalent fraction of these cells were KLRG1^+^ CD127^lo^ SLEC not only at day 8 but also at day 30, when the LCMV response is in the effector or effector-memory stage (Figure 2H; Figure S2 in Supplementary Material). These findings indicate that the influence of GPR18 on establishment of a KLRG1^+^ CD8 cell compartment varies depending on the induction conditions.

**Figure 3:**
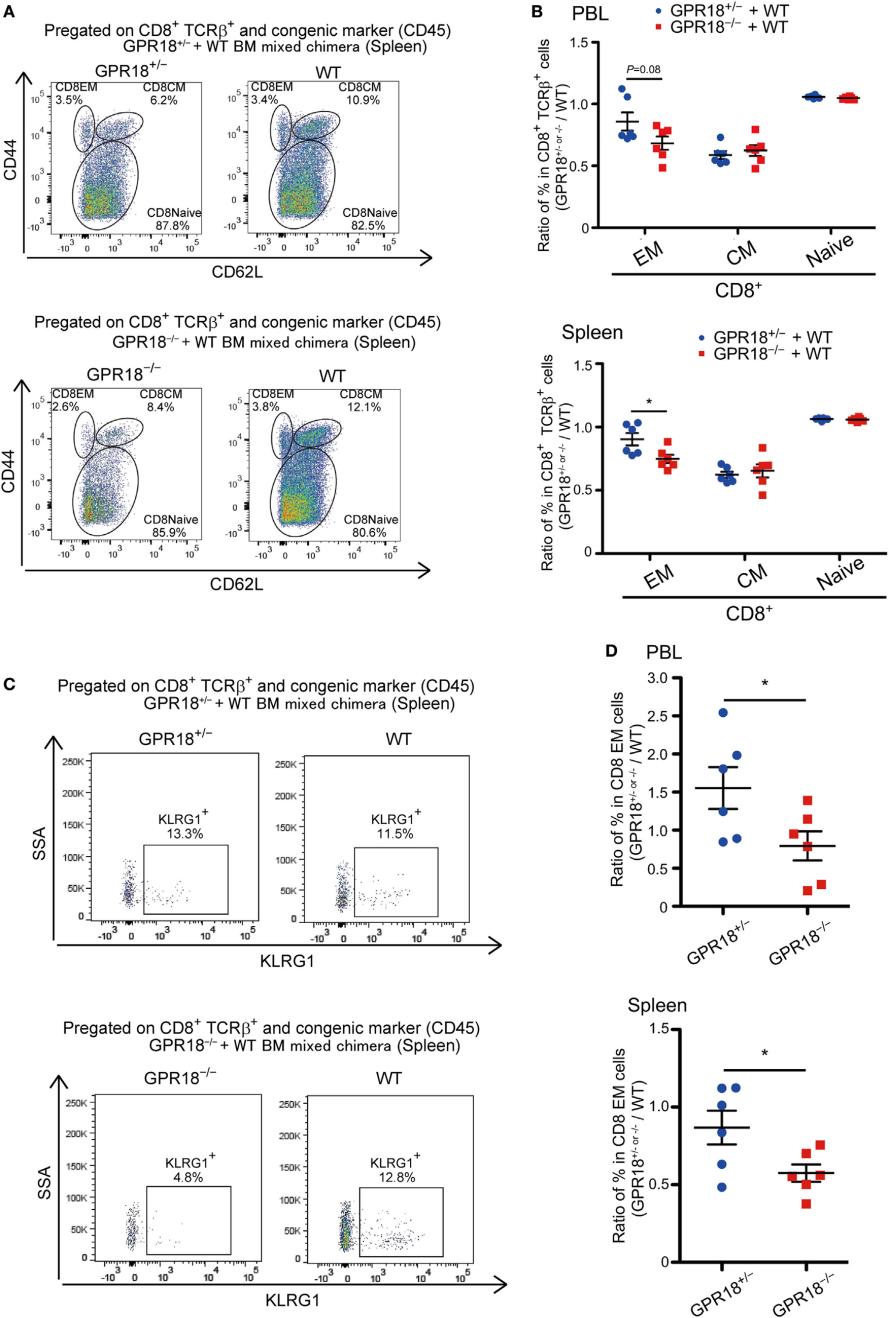
Cell intrinsic defects of KLRG1^+^ CD8 T cells in G-protein coupled receptor 18 (GPR18)-deficiency. B6-CD45.1^+^ mice were reconstituted with CD45.1/2^+^ WT and CD45.2^+^ *Gpr18*^+/^*^−^* or *Gpr18^−/−^* bone marrow (BM) 10 weeks before analysis (*n* = 6). **(A)** Flow cytometric analysis of CD44 and CD62L expression in CD8^+^ TCRβ^+^ and congenic (CD45) marker^+^ splenocytes from the indicated donor cells in the same animal. Numbers show percentage of cells in the indicated gate. **(B)** Naïve (CD44^lo^CD62L^hi^), effector memory (EM) (CD44^hi^CD62L^lo^), and CM (CD44^hi^CD62L^hi^) T cells in *Gpr18*^+/^*^−^* or *Gpr18^−/−^* CD8^+^ TCRβ^+^ cells, identified as in (A), were presented as a ratio to the WT control donor cells from the same animal in peripheral blood lymphocytes (PBL) (upper panel) or spleen (lower panel). **(C)** Flow cytometric analysis of KLRG1 expression in CD8 EM splenocytes from the indicated donor cells in the same animal. **(D)** Percentage of KLRG1^+^ cells in *Gpr18*^+/^*^−^* or *Gpr18^−/−^* CD8^+^ EM TCRβ^+^ cells, determined as in **(C)**, presented as a ratio to the WT control donor cells from the same animal in PBL (upper panel) or spleen (lower panel). Each symbol in **(B,D)** represents an individual mouse, and lines represent means ± SEM. *p < 0.05 by Student’s *t*-test **(B,D)**. Data from one of two or three independent experiments are shown.

To determine if the reduction in CD8 EM reflected a cell intrinsic role for GPR18, we generated mixed BM chimeras. Analysis of these mice 10 weeks after reconstitution revealed a selective deficiency in GPR18 KO EM cells in blood and spleen (Figures 3A,B). The effect was again most prominent for KLRG1^+^ CD8 EM cells (Figures 3C,D). These data indicate that the GPR18 receptor acts intrinsically to favor establishment or maintenance of the KLRG1^+^ CD8 EM compartment.

**Figure 4:**
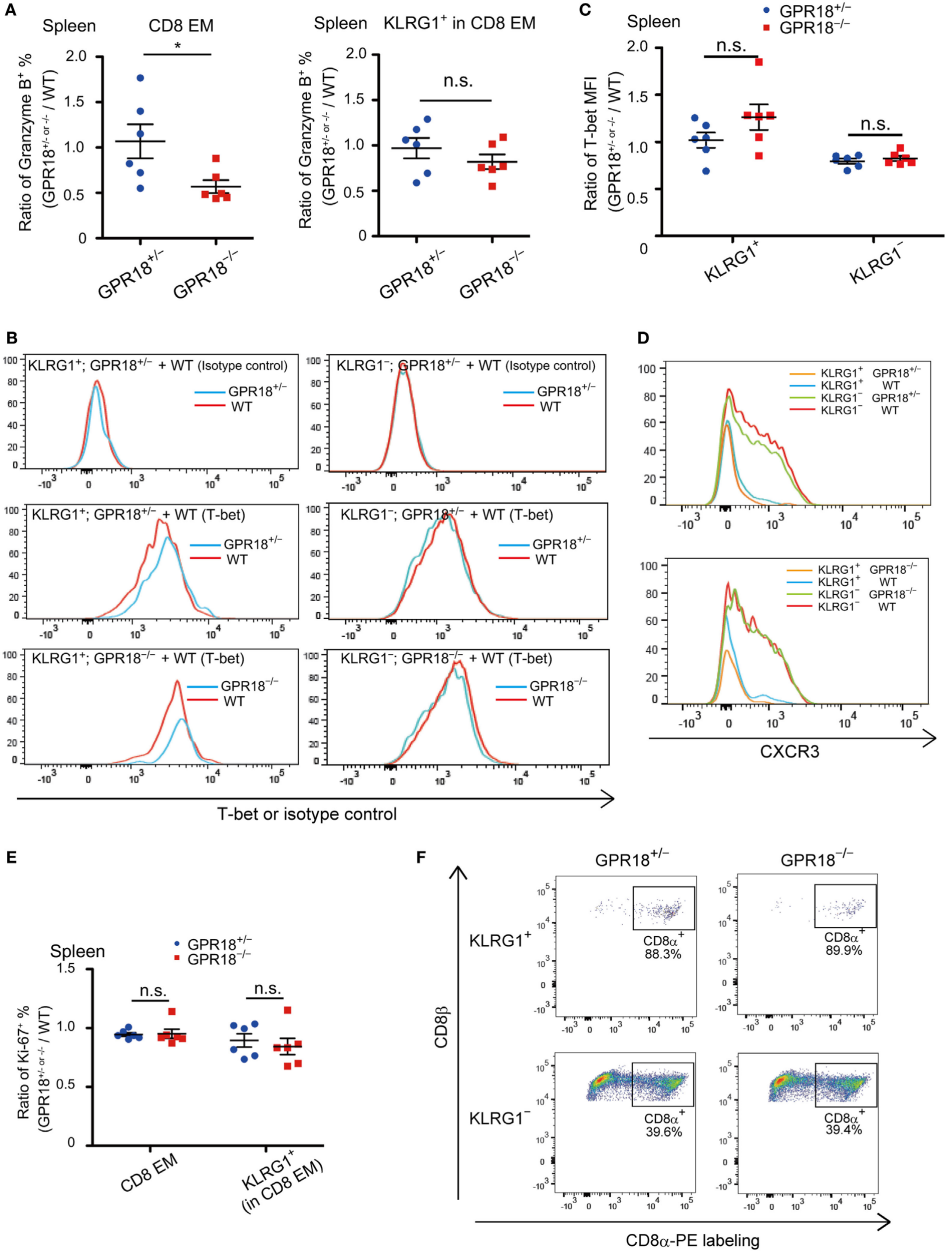
Granzyme B, T-bet, CXCR3, and Ki67 expression in *Gpr18^−/−^* CD8 effector memory (EM) cells. **(A)** Intracellular staining of splenic CD8 EM (left panel) or KLRG1^+^ (right panel) cells for Granzyme B. Data are plotted as ratio of Granzyme B^+^ *Gpr18*^+/−^ or *Gpr18^−/−^* cells and WT cells in the same mixed bone marrow (BM) chimeric animal. **(B)** Representative histograms of T-bet staining in KLRG1^+^ (left panels) or KLRG1*^−^* (right panels) CD8 EM splenocytes. Upper panels, stained with isotype control antibodies, middle and lower panels stained with T-bet antibodies. Cells were from mixed BM chimeras of the indicated types. *y-*axis of histogram overlays was normalized to mode. **(C)** Mean fluorescence intensity (MFI) of T-bet staining in KLRG1^+^ or KLRG1*^−^* CD8 EM splenocytes, plotted as ratio of MFI in *Gpr18^+/−^* or *Gpr18^−/−^* compared to control WT in mixed BM chimeras (*n* = 6). **(D)** Flow cytometry analysis of CXCR3 expression in KLRG1^+^ and KLRG1*^−^* CD8 EM cells from mixed BM chimeras. Upper panel; histograms from *Gpr18*^+/−^ plus WT mixed BM chimera. Lower panel; histograms from *Gpr18^−/−^* plus WT mixed BM chimera. *y-*axis of histogram overlays was normalized to mode. **(E)** Ratio of percentage of Ki67^+^ CD8 EM or KLRG1^+^ CD8 EM cells in *Gpr18*^+/−^ or *Gpr18^−/−^* compared to control WT in mixed BM chimeras (*n* = 6). **(F)** *In vivo* labeling with CD8α-PE antibody. Three minutes after antibody injection into the indicated mice, splenocytes were harvested and stained *ex vivo* with antibodies to identify KLRG1^+^ or KLRG1^−^ CD8 EM cells. Representative plots gated on KLRG1^+^ (upper panels) and KLRG1^−^ (lower panels) are shown. Numbers show percentage of cells in indicated CD8α-PE^+^ gate. Each symbol in **(A,C,E)** represents an individual mouse, and lines represent means ± SEM. *p < 0.05, “n.s.” *p* > 0.05 by Student’s *t*-test **(A,C,E)**. Data from one of two independent experiments are shown.

To rule out that the reduction in CD8 EM cells was due to selective loss of the KLRG1 surface marker, we stained CD8 T cells in mixed BM chimeras for Granzyme B, since this gene is highly expressed in KLRG1^+^ cells (2, 3). The frequency of Granzyme B^+^ cells was reduced in GPR18 KO CD8 EM to an extent similar to the reduction in KLRG1^+^ cells (Figure 4A, left panel and Figure 3A; Figure S3A in Supplementary Material). When gating on the KLRG1^+^ population, the fraction of Granzyme B^+^ cells was not altered, in accord with a reduction in population size rather than selective changes in marker expression (Figure 4A right panel; Figure S3B in Supplementary Material). Since T-bet (Tbx21) is needed for KLRG1^+^ CD8 EM cell development, we tested whether its expression was affected by GPR18 deficiency. Intracellular flow cytometry showed similar expression in the KLRG1^+^ CD8 cells that were present in GPR18 KO mice compared to those present in matched controls (Figures 4B,C). Indeed, rather than being reduced, a non-statistically significant trend for increased T-bet expression was observed. T-bet expression levels in KLRG1^−^ cells were lower than in KLRG1^+^ cells as expected and were equivalent in KO and control (Figures 4B,C). These data indicate that GPR18 is not required for upregulation of the T-bet transcription factor in CD8 EM cells.

CXCR3 affects the balance between effector and memory CD8 T-cell generation (19), and we, therefore, investigated expression levels of this chemokine receptor. CXCR3 was expressed comparably in GPR18 KO and wild-type CD8 T cells (Figure 4D). Further analysis of the mixed BM chimeras showed that the fraction of cells that were in or had recently been in cell cycle, as determined by Ki-67 staining, was unaltered by GPR18-deficiency (Figure 4E; Figures S4A,B in Supplementary Material). IFNγ staining showed that expression of this cytokine in CD8 cells was not affected by GPR18 deficiency (Figure S5 in Supplementary Material).

Since KLRG1^+^ and KLRG1^−^ effector-memory cells differ in their distribution within the spleen, with KLRG1^+^ cells locating predominantly in the highly vascular red pulp (9, 20), we tested for any effect of GPR18 deficiency on cell distribution using *in vivo* CD8α-PE labeling. This technique labels cells that are in blood-exposed compartments such as the red pulp while leaving cells in the lymphoid-rich white pulp unlabeled (18, 21). As expected, KLRG1^−^ cells were predominantly protected from labeling, being enriched in the white pulp, while KLRG1^+^ cells were highly labeled (Figure 4F). GPR18-deficiency did not affect the fraction of KLRG1^−^ or KLRG1^+^ cells that were labeled, indicating that the cells were located in their correct compartments.

**Figure 5:**
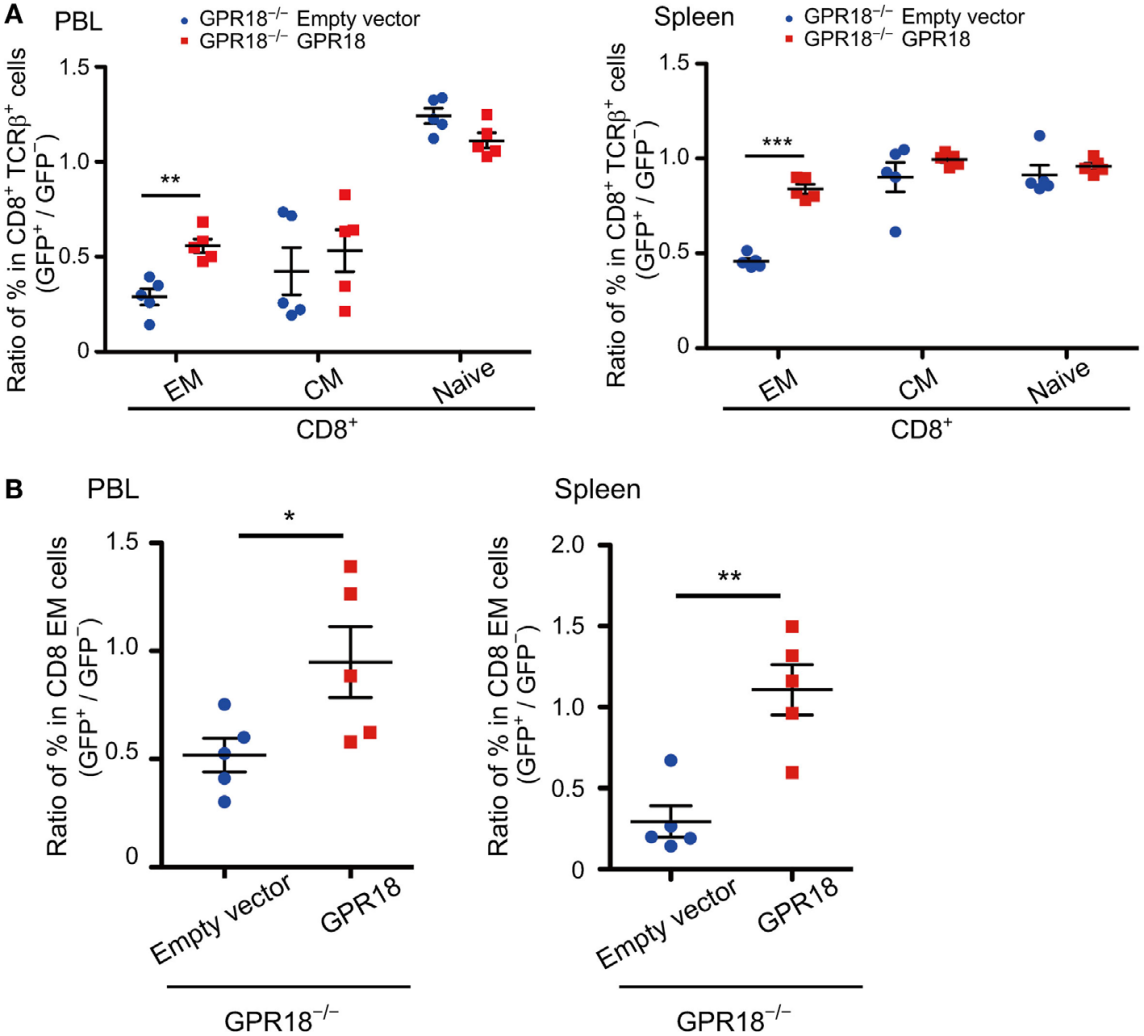
Rescue effects of G-protein-coupled receptor 18 (GPR18) expression on CD8 effector memory (EM) cells in *Gpr18^−/−^* mice. **(A,B)** CD45.2^+^ *Gpr18^−/−^* bone marrow transduced with empty vector or Gpr18 retrovirus was used to reconstitute CD45.1^+^ recipients (*n* = 5). Donor cells were gated on CD45.1^−^CD45.2^+^ and then gated on GFP^+^ (transduced donor cells) or GFP^−^ (untransduced donor cells). **(A)** CD8^+^ TCRβ^+^ cells from peripheral blood lymphocytes (PBL) (left panel) or spleen (right panel) were stained for naïve (CD44^lo^CD62L^hi^), EM (CD44^hi^CD62L^lo^), and CM (CD44^hi^CD62L^hi^). Percentage of EM, CM, and naïve populations in GFP^+^ donor cells were presented as a ratio to those in GFP^−^ donor cells from the same animal. **(B)** CD8 EM cells from PBL (left panel) or spleen (right panel) were stained for KLRG1. Percentage of KLRG1^+^ populations in GFP^+^ donor cells were presented as a ratio to those in GFP^−^ donor cells from the same animal. Each symbol represents an individual mouse, and lines represent means ± SEM. ***p < 0.001, **p < 0.01, *p < 0.05 by Student’s *t*-test.

Finally, to confirm that the phenotype observed was solely due to GPR18 deficiency, we restored GPR18 expression by transduction of GPR18 KO BM cells with a GPR18 and GFP encoding retrovirus, versus a GFP control retrovirus (empty vector). Irradiated mice were reconstituted with the transduced BM cells and then analyzed for the frequency of each CD8 cell type that was transduced (GFP^+^) versus untransduced (GFP^−^) to test for enrichment or depletion of the transduced cells. Compared to mice reconstituted with GPR18 KO BM transduced with the empty vector, mice receiving GPR18 KO BM transduced with GPR18 showed a selective increase in transduced CD8 EM and KLRG1^+^ cells in blood and spleen (Figures 5A,B; Figures S6A,B in Supplementary Material). These data provide strong support for the conclusion that the altered CD8 T cell compartments in the GPR18-deficient mice reflects a direct requirement for GPR18.

## Discussion

Our findings establish a cell intrinsic role for GPR18 in the normal accumulation of CD8 effector T cells, in particular, the KLRG1^+^ EM cell population. The CD8 effector cells studied here are those arising endogenously over time in mice housed in a specific pathogen-free colony. In experiments where we infected mice with the pathogen LCMV (Armstrong strain), we did not detect clear differences in the induced EM cell populations between GPR18 KO and littermate control mice. Further studies will be needed to define the types of response where GPR18 contributes to KLRG1^+^ CD8 EM cell development or maintenance, but at this time, we suggest the receptor is influencing their development in response to commensal microorganisms. Two ligands have been proposed for GPR18, *N*-arachidonyl glycine (NAGly), and resolvin-D2 (22–24). In our previous work and studies by others, NAGly has not been confirmed to be a functional GPR18 agonist (14, 25). Future studies will be needed to determine whether resolvin-D2 or an as yet unidentified GPR18 ligand acts to promote effector CD8 T cell homeostasis. Our studies suggest that small molecule agonists of GPR18 might augment the size of the KLRG1^+^ effector CD8 T cell compartment, an effect that might be beneficial, for example, during viral responses or in the context of tumor immunotherapy. Our findings may also prove significant for understanding the GPR18 SNPs detected as being enriched in inflammatory bowel disease patients in genome-wide association studies (26, 27).

## Ethics Statement

All experiments conformed to ethical principles and guidelines approved by the UCSF Institutional Animal Care and Use Committee.

## Author Contributions

HS performed experiments and analyzed the data. HS and JC designed experiments, interpreted the data, and wrote the manuscript.

## Conflict of Interest Statement

The authors declare that the research was conducted in the absence of any commercial or financial relationships that could be construed as a potential conflict of interest.

## Supplementary Material

Figure S1 Comparable CD4 T cells in G-protein-coupled receptor 18 (GPR18)-deficient mice. **(A)** Flow cytometric analysis of CD44 and CD62L expression in CD4^+^ TCRβ^+^ peripheral blood lymphocytes (PBL) from the indicated mature (6 months old) mice. Numbers show percentage of cells in the indicated gate. **(B)** Number of CD4^+^ TCR β^+^ splenocytes in the indicated mature (6 months old) mice. Each population was pre-gated on CD45^+^TCRβ^+^ cells. *Gpr18^+/−^, n* = 12; *Gpr18^−/−^, n* = 12.

Figure S2 Comparable CD8 effector memory and short-lived effector cells (SLEC) in *Gpr18^−/−^* mice after infection with lymphocytic choriomeningitis virus (LCMV) Armstrong. Representative flow cytometric plots and gating strategy for Figures 2G,H. peripheral blood lymphocytes (PBL) from *Gpr18^+/−^* (upper panels) or *Gpr18^−/−^* (lower panels) at day 30 after LCMV Armstrong infection.

Figure S3 Granzyme B expression in CD8 effector memory (EM) and KLRG1^+^ cells in *Gpr18^−/−^* mice. **(A,B)** Representative flow cytometric plots for Figure 4A for splenocytes from the indicated donor cells in the same animal [*Gpr18^+/−^* (left) or *Gpr18^−/−^* (right) compared to control WT in mixed bone marrow (BM) chimeras]. **(A)** CD8 EM cells and **(B)** short-lived effector cells (SLEC). Numbers show percentage of cells in the indicated gate.

Figure S4 Comparable percentage of Ki-67^+^ CD8 effector memory (EM) or short-lived effector cells in *Gpr18^−/−^* mice. **(A,B)** Representative flow cytometric plots for Figure 4C for splenocytes from the indicated donor cells in the same animal [*Gpr18^+/−^* (left) or *Gpr18^−/−^* (right) compared to control WT in mixed bone marrow (BM) chimeras]. **(A)** CD8 EM cells and **(B)** KLRG1^+^ cells. Numbers show percentage of cells in the indicated gate.

Figure S5 Comparable IFN-γ production in *Gpr18^−/−^* and control CD8 T cells. Intracellular staining of CD8 effector memory (EM) splenocytes for IFN-γ, shown as percentage of IFN-γ producing cells from *Gpr18^+/−^* or *Gpr18^−/−^* mice after 5 h *in vitro* stimulation with phorbol myristate acetate plus ionomycin. *Gpr18^+/−^, n* = 4; *Gpr18^−/−^, n* = 5. Combined data from two independent experiments are shown.

Figure S6 Rescue effect of G-protein coupled receptor 18 (GPR18) expression on CD8 effector memory (EM) and KLRG1^+^ cells in *Gpr18^−/−^* mice. **(A,B)** Gating strategy for Figure 5 for peripheral blood lymphocytes (PBL) from empty vector-transduced GPR18^−/−^ bone marrow (BM) chimera mice **(A)** or GPR18-transduced GPR18*^−^*^/^*^−^* BM chimera mice **(B)**. Numbers show percentage of cells in the indicated gate.
