# Supplementary figures and images for: G-Protein Coupled Receptor 18 Contributes to Establishment of the CD8 Effector T Cell Compartment
Source: Front Immunol. 2018 Apr 4;9:660. doi: 10.3389/fimmu.2018.00660 (PMC5893653; doi:10.3389/fimmu.2018.00660)

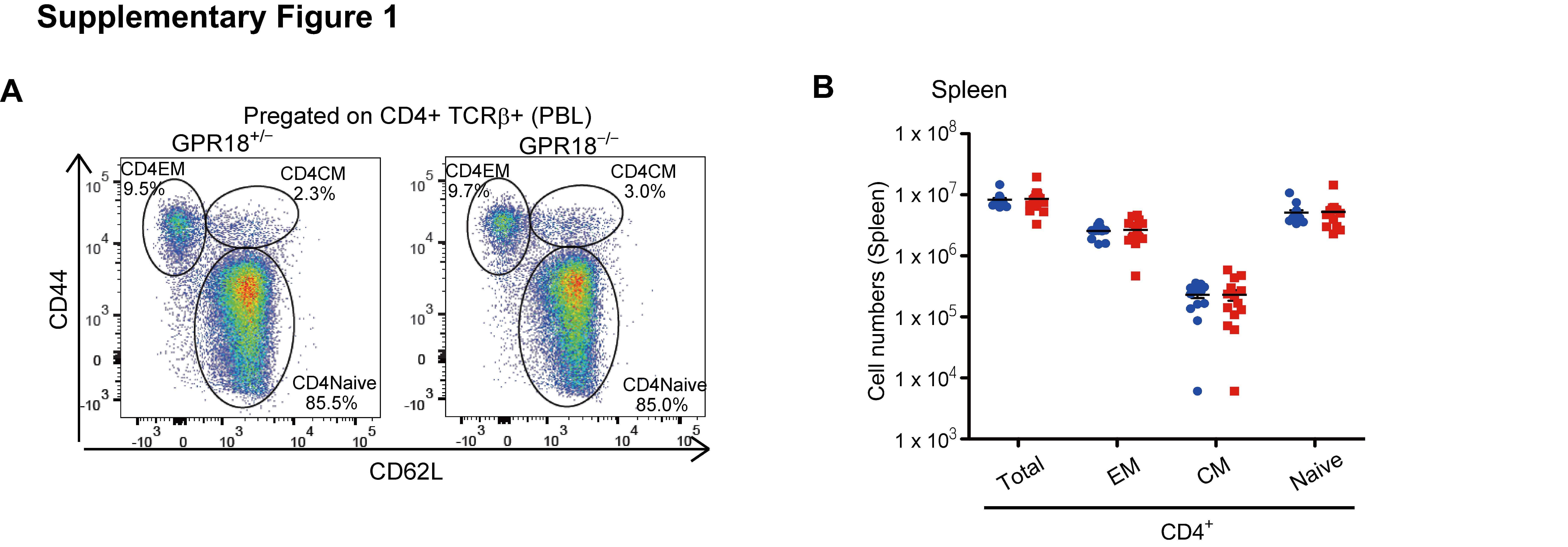

Supplement: Figure S1 — Comparable CD4 T cells in G-protein-coupled receptor 18 (GPR18)-deficient mice. (A) Flow cytometric analysis of CD44 and CD62L expression in CD4+ TCRβ+ peripheral blood lymphocytes (PBL) from the indicated mature (6 months old) mice. Numbers show percentage of cells in the indicated gate. (B) Number of CD4+ TCR β+ splenocytes in the indicated mature (6 months old) mice. Each population was pre-gated on CD45+TCRβ+ cells. Gpr18+/−, n = 12; Gpr18−/−, n = 12. [file image_1.tif]

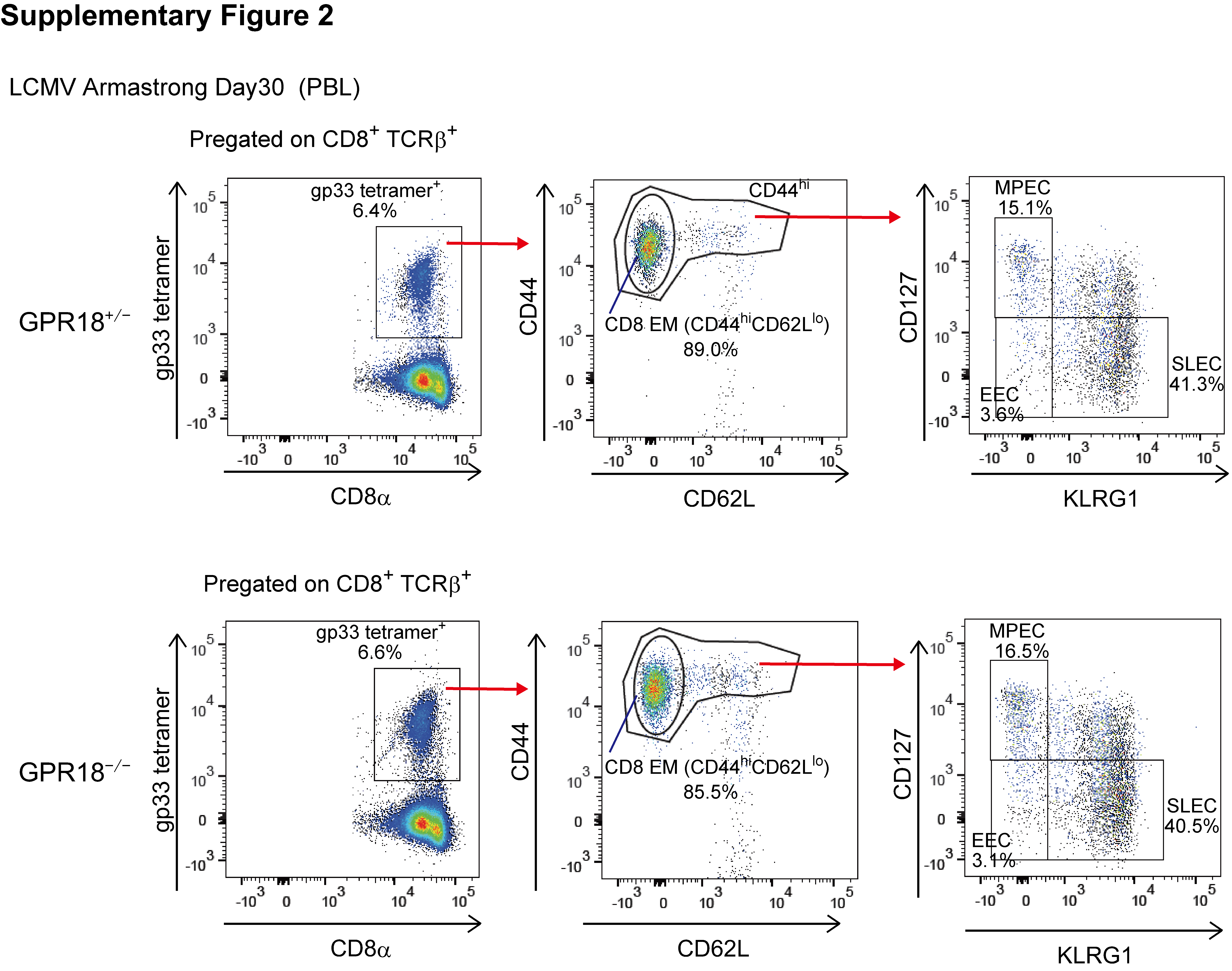

Supplement: Figure S2 — Comparable CD8 effector memory and short-lived effector cells (SLEC) in Gpr18−/− mice after infection with lymphocytic choriomeningitis virus (LCMV) Armstrong. Representative flow cytometric plots and gating strategy for Figures 2G,H. peripheral blood lymphocytes (PBL) from Gpr18+/− (upper panels) or Gpr18−/− (lower panels) at day 30 after LCMV Armstrong infection. [file image_2.tif]

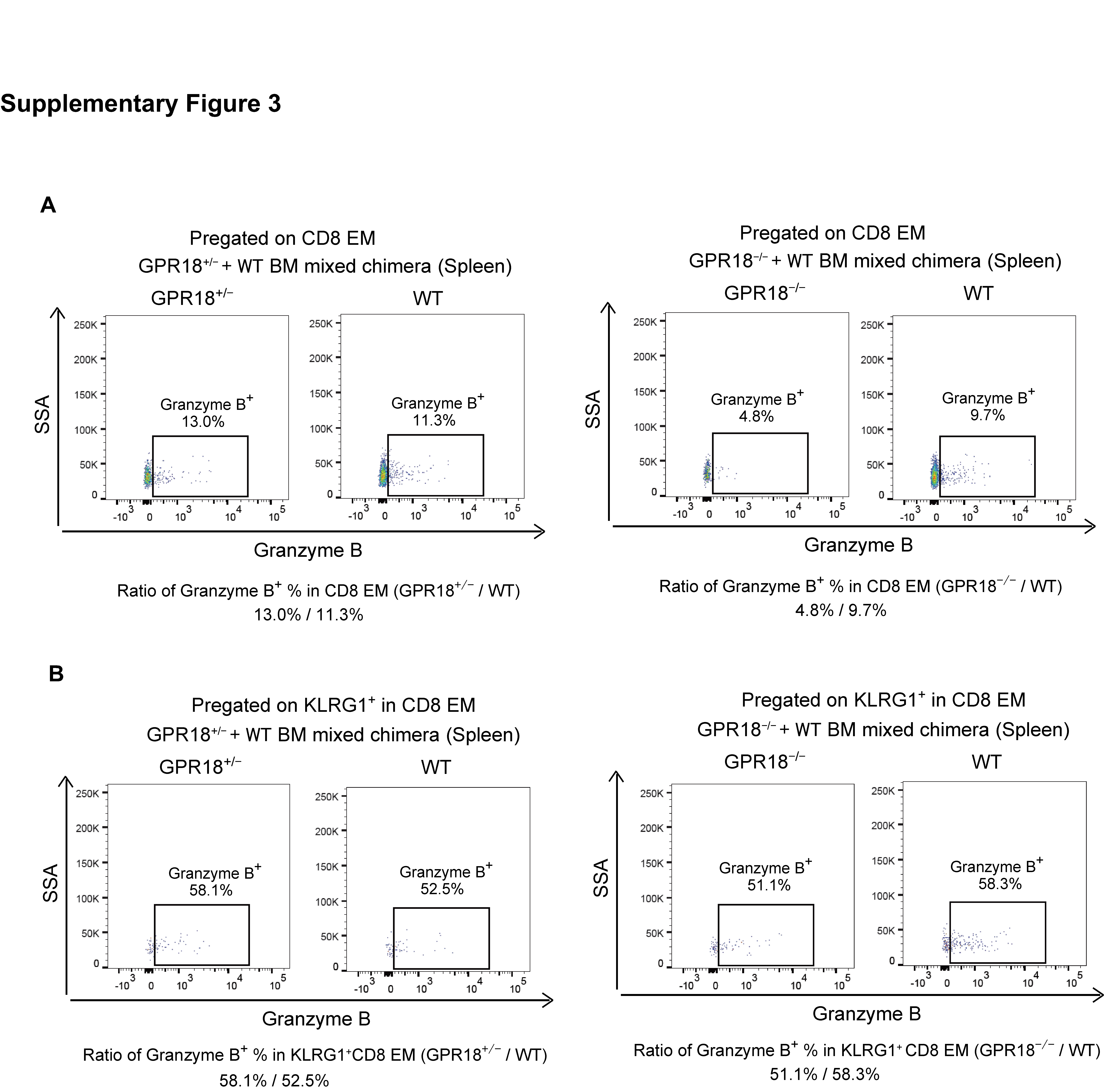

Supplement: Figure S3 — Granzyme B expression in CD8 effector memory (EM) and KLRG1+ cells in Gpr18−/− mice. (A,B) Representative flow cytometric plots for Figure 4A for splenocytes from the indicated donor cells in the same animal [Gpr18+/− (left) or Gpr18−/− (right) compared to control WT in mixed bone marrow (BM) chimeras]. (A) CD8 EM cells and (B) short-lived effector cells (SLEC). Numbers show percentage of cells in the indicated gate. [file image_3.tif]

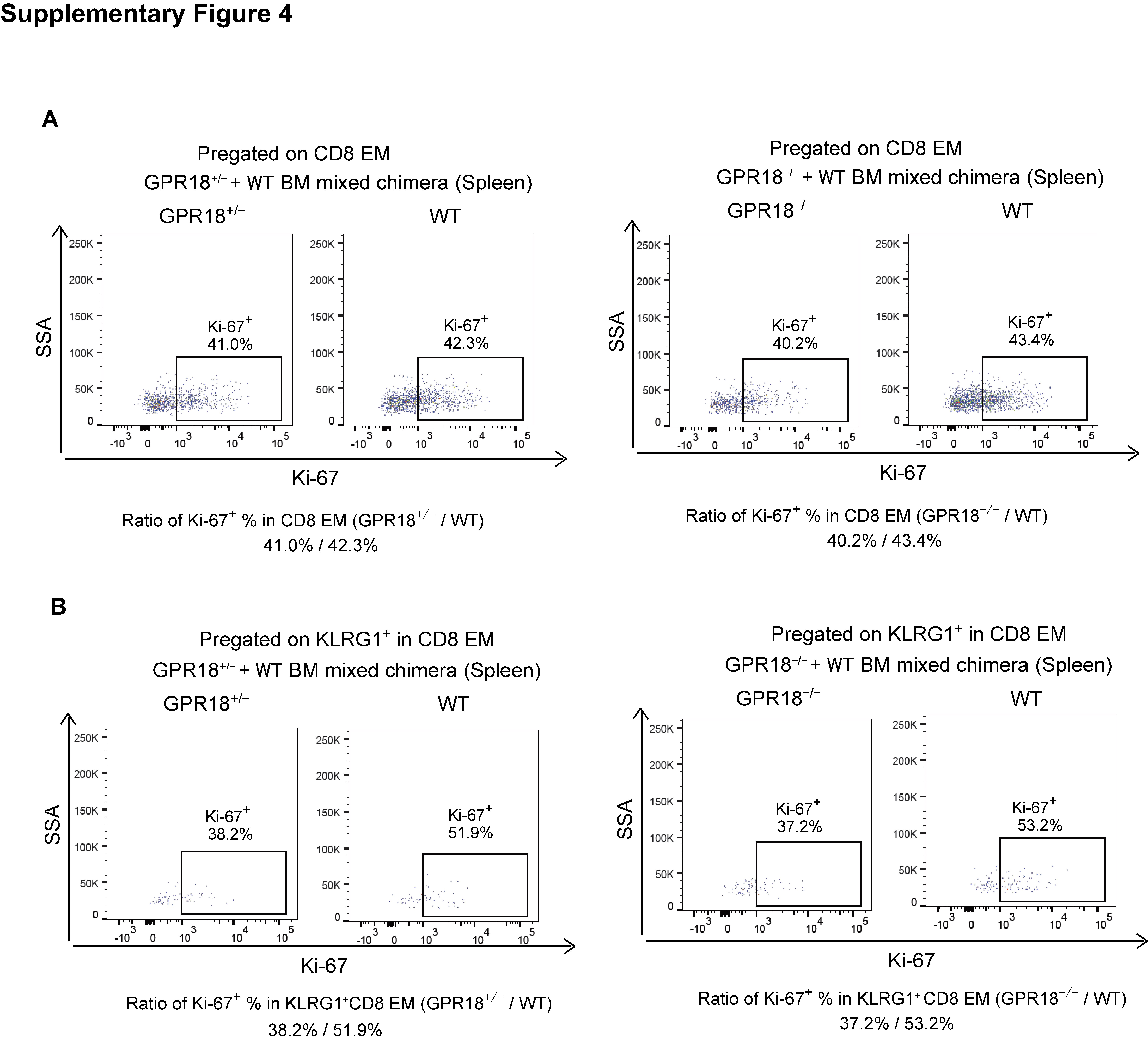

Supplement: Figure S4 — Comparable percentage of Ki-67+ CD8 effector memory (EM) or short-lived effector cells in Gpr18−/− mice. (A,B) Representative flow cytometric plots for Figure 4C for splenocytes from the indicated donor cells in the same animal [Gpr18+/− (left) or Gpr18−/− (right) compared to control WT in mixed bone marrow (BM) chimeras]. (A) CD8 EM cells and (B) KLRG1+ cells. Numbers show percentage of cells in the indicated gate. [file image_4.tif]

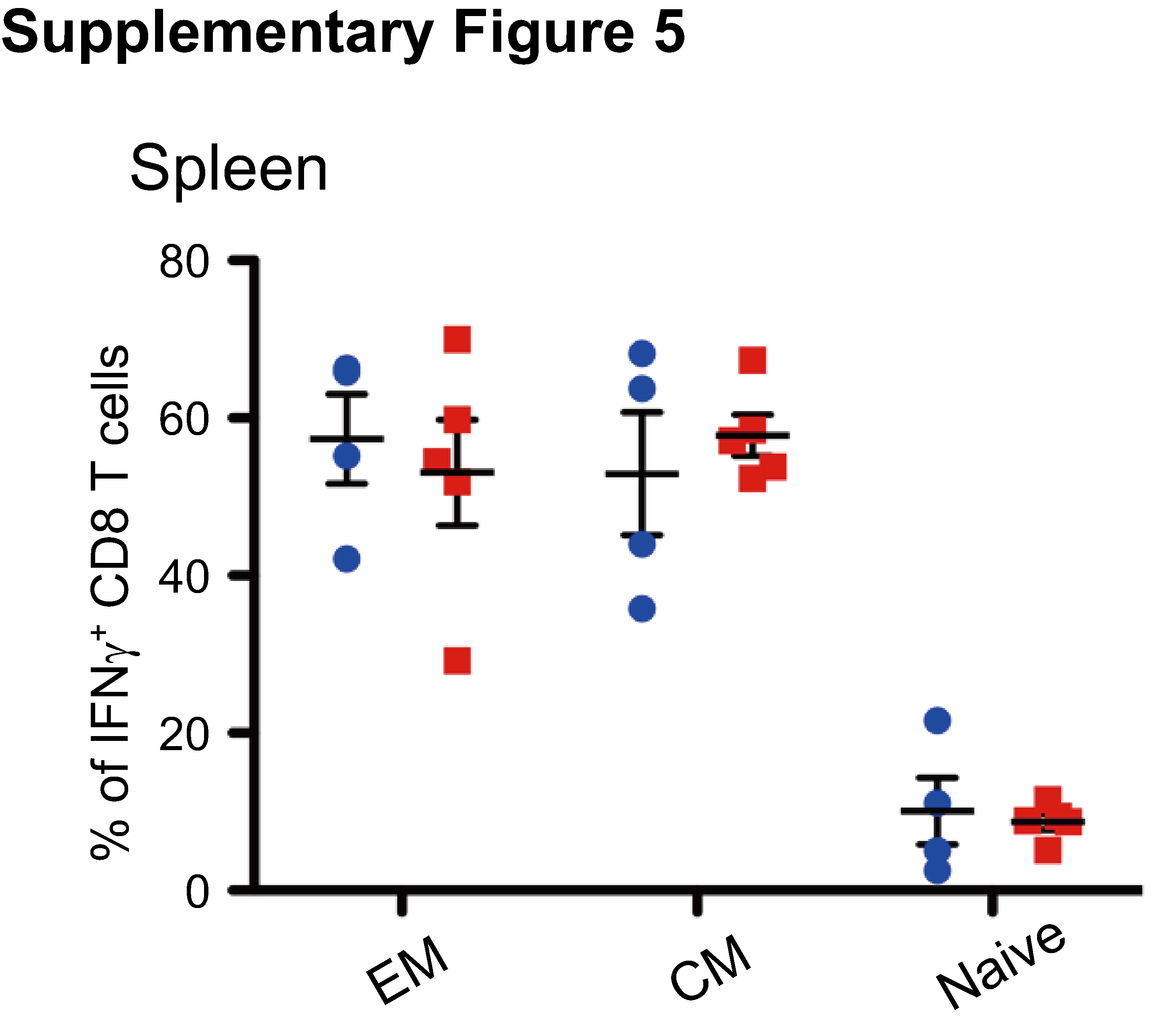

Supplement: Figure S5 — Comparable IFN-γ production in Gpr18−/− and control CD8 T cells. Intracellular staining of CD8 effector memory (EM) splenocytes for IFN-γ, shown as percentage of IFN-γ producing cells from Gpr18+/− or Gpr18−/− mice after 5 h in vitro stimulation with phorbol myristate acetate plus ionomycin. Gpr18+/−, n = 4; Gpr18−/−, n = 5. Combined data from two independent experiments are shown. [file image_5.tif]

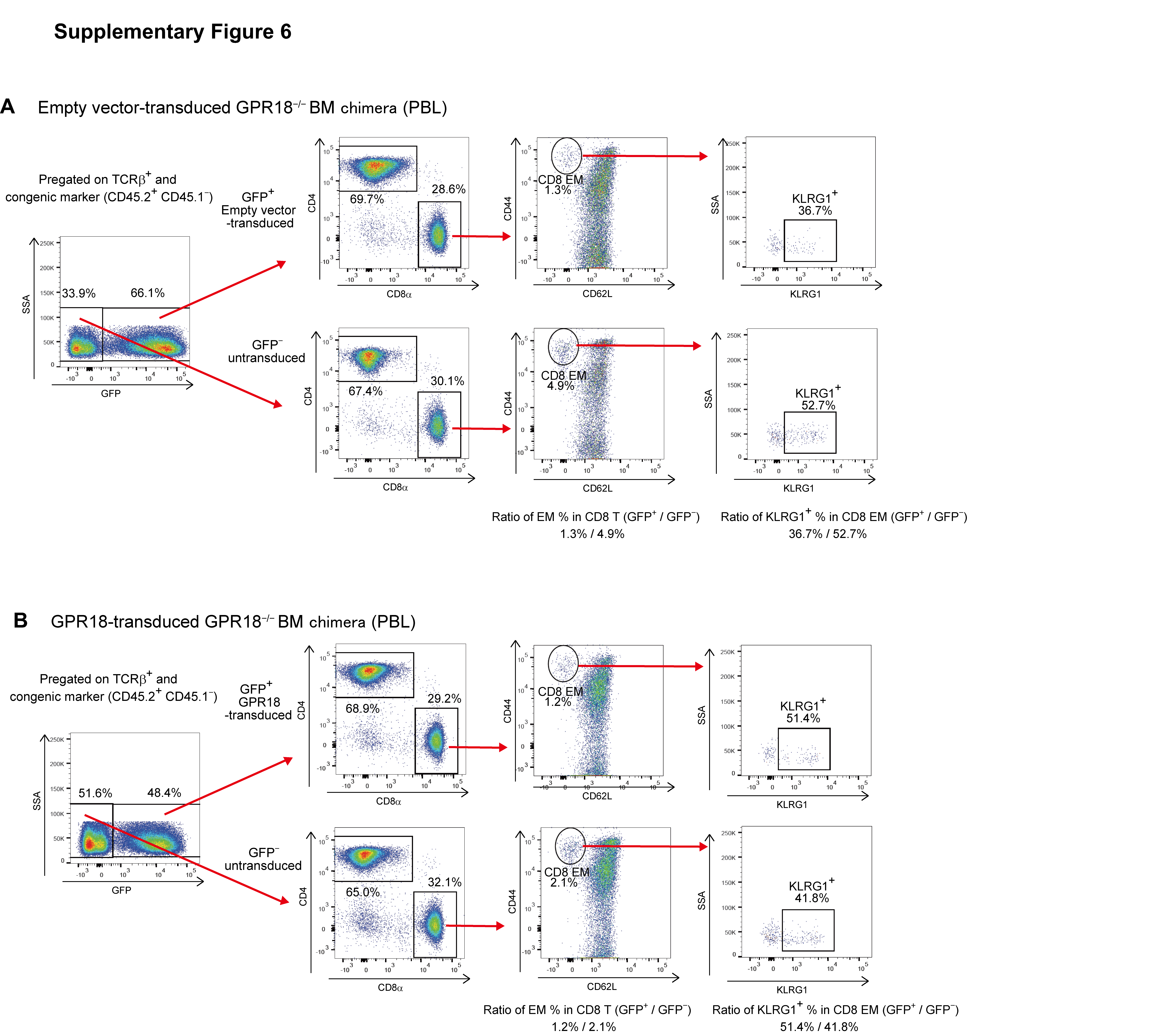

Supplement: Figure S6 — Rescue effect of G-protein coupled receptor 18 (GPR18) expression on CD8 effector memory (EM) and KLRG1+ cells in Gpr18−/− mice. (A,B) Gating strategy for Figure 5 for peripheral blood lymphocytes (PBL) from empty vector-transduced GPR18−/− bone marrow (BM) chimera mice (A) or GPR18-transduced GPR18−/− BM chimera mice (B). Numbers show percentage of cells in the indicated gate. [file image_6.tif]
